# Is It a Challenge to Use Molecular Hydrogen for Extending Flower Vase Life?

**DOI:** 10.3390/plants11101277

**Published:** 2022-05-10

**Authors:** Toan Khac Nguyen, Jin Hee Lim

**Affiliations:** Department of Plant Biotechnology, Sejong University, Seoul 05006, Korea; toanchrys@sju.ac.kr

**Keywords:** cut flower, flower industry, postharvest quality, postharvest technique, the fourth industrial revolution, vase life

## Abstract

Currently, molecular hydrogen treatment has the potential to manage the Corona Virus disease (COVID-19) and pandemic based on its anti-inflammatory, apoptosis-resistance, antioxidant, and hormone-regulating properties. Antioxidant properties are beneficial in both animal and human diseases. In agricultural sciences, molecular hydrogen is used to postpone postharvest ripening and senescence in fruits. However, studies on flower senescence are limited to the application of hydrogen molecules during floral preharvest and postharvest. Fortunately, improved tools involving molecular hydrogen can potentially improve postharvest products and storage. We also discuss the benefits and drawbacks of molecular hydrogen in floral preharvest and postharvest. This review provides an overview of molecular hydrogen solutions for floral preservative storage.

## 1. Introduction

The floral market is defined by the high quality of the commercial standard pipeline from floral farmers to the final customers. The flowers, which are considered beautiful symbols of love, ceremony, appreciation, and respect, undergo discoloration, bending, or shriveling in commercialized sectors, reducing the quality of the floral products. Preharvest, vase life, and postharvest values are the most important characteristics for evaluating the commercial quality of cut flowers [1,2]. The preharvest quality of cut flowers is affected by temperature and seasonal variations [2]. Postharvest quality is influenced by multiple genetic factors, conditions of the preharvest environment [3], postharvest management practices throughout the supply chain [4], plant maturity, planting and harvesting seasons [5], nutritional status [6], water balance, and postharvest temperature [7]. Vase life and cut flower quality can be improved by evaluating appropriate genotypes in breeding programs [7,8,9], selecting the optimal temperature for storage and transport [10], and applying exogenous chemical substances such as sucrose [11], salicylic acid, glutamine [12], gibberellic acid [13], humic acid [14], and 1-methylcyclopropene [15].

**Figure 1:**
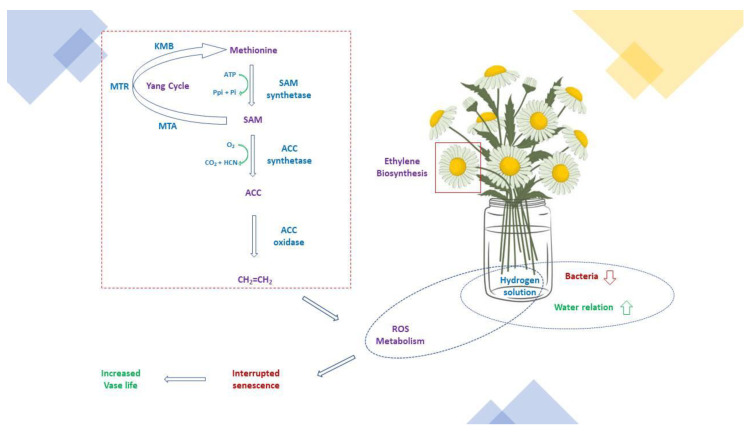
Possible roles of the effective hydrogen solution in floral preservative solution.

To date, the COVID-19 pandemic has been restricted by vaccination and it can be potentially achieved by molecular hydrogen treatment through its characteristics, such as apoptosis-resistance, antioxidant, anti-inflammatory, and hormone-regulating properties. In agricultural sciences, molecular hydrogen is applied to postpone postharvest ripening and senescence in fruits. Hydrogen and its forms are well-known power energy carriers with numerous applications, and they can be easily transported. Hydrogen gas (H_2_) has a broad range of biological effects and is a useful tool in medicine and agriculture [16,17,18,19]. H_2_ affects plant growth, stress-tolerance enhancement [17], and possesses important effects on bacteria communities by preventing bacterial blockage and rot in xylem vessels [20]. H_2_ has beneficial effects and improves the vase life quality of cut flowers, such as roses, by enhancing the beneficial bacteria abundances present on the stem-end cut surface [20]. The postharvest senescence of cut flowers results in significant commercial production losses, which are linked to a series of signaling molecules, such as magnesium hydride (MgH_2_) with H_2_-releasing material [21], ethylene [22], ROS [23] (Figure 1), and nitric oxide (NO) [24]. Recently, the application of H_2_ in the form of hydrogen-rich water (HRW) was shown to delay postharvest senescence and increase the quality of cut flowers [25,26,27]. H_2_ can inhibit ethylene roles and affects signal transduction to regulate the expression of related genes (*Rh-ACS3*, *Rh-ACO1*, and *Rh-ETR1*), thus delaying flower senescence during the vase period [27]. In addition, H_2_-stimulated NO can act as a downstream signaling molecule to maintain postharvest quality in cut lilies [28]. In this review, we discuss the benefits and drawbacks of molecular hydrogen in floral postharvest periods. This study considers the application of molecular hydrogen tools in floral preservation.

## 2. The Impact of Hydrogen Solution in Floral Preharvest and Postharvest

In roses, the quality of vase life is important for supporting innovative solutions that improve postharvest techniques [29]. In cut roses (*Rosa hybrida* ‘Movie star’), the study of HRW showed a significantly extended vase life of cut roses by regulating the bacteria community of the stem ends [20]. HRW inhibited the bacterial blockages caused by bacteria colonization and biofilm formation in rose xylem vessels [20]. Therefore, it increased water uptake and extended cut rose vase life. By using high-throughput sequencing of the 16S rRNA gene sequence, it was concluded that HRW significantly developed the richness of bacterial communication on the stem-end cut surface [20]. The beneficial abundances were developed by 1% HRW on the stem-end cut surface, and it can be a key factor for prolonging flower vase life, especially in roses [20]. In another cut rose (*Rosa hybrida* ‘Carola’), the study of H_2_-releasing materials, such as MgH_2_-treated cut rose flowers, is shown as an alternative tool for a more flexible and convenient hydrogen supply [21]. The effect of 0.001 g L^−1^ MgH_2_-treated cut rose flowers was related to that of 10% HRW produced by electrolysis (similarly hereinafter) [21]. This study validated a critical role for the stimulated NO in the MgH_2_-extended vase life of cut flowers [21].

In cut lily (*Lilium* spp.) flowers, treatment with HRW at 0.5% and 1% increased vase life and maintained maximum flower diameter [25]. In cut rose (*Rosa hybrid* L.) flowers, 50% HRW treatment significantly extended vase life and provided the maximum flower diameter [25]. The leaf relative water content and fresh weight of cut lilies and roses were improved by appropriate doses of HRW [25]. Compared with the control, the leaf stomata size was diminished in cut lily and rose flowers in the HRW treatment [25]. HRW treatment significantly decreased leaf MDA content, and reduced electrolyte leakage in cut lilies [25]. Both cut lily and rose flowers showed improved antioxidant enzyme activities [25]. Exogenously applied H_2_ might increase vase life and improve postharvest quality in cut flowers by controlling water balance and membrane stability, and by reducing stomata size and oxidative damage [25].

In cut lilies (*Lilium* ‘Manissa’), the relationship between H_2_ and NO was studied, and differentially accumulated proteins were identified during postharvest freshness [28]. HRW (1%) and 150 μM sodium nitroprusside (SNP) significantly improved vase life and quality, whereas NO inhibitors suppressed the positive effects of HRW [28]. Proteomic analysis showed 50 differentially accumulated proteins in lily leaves, which were divided into seven functional categories [28]. Among them, ATP synthase CF1 alpha subunit (chloroplast) (AtpA) was up-regulated by HRW and down-regulated by the NO inhibitor [28]. NO might be affected by H_2_-improved freshness of cut lilies, and the AtpA protein can play a critical role during this process [28].

Hydrogen nanobubble water (HNW) was used to screen cut carnation flowers (*Dianthus caryophyllus* L.) for delayed senescence [30]. Compared to conventional HRW, HNW had higher concentration properties and residence times for dissolved hydrogen gas [30]. The application of 5% HNW significantly increased the cut carnation vase life compared with distilled water, other doses of HNW (including 1%, 10%, and 50%), and 10% HRW, which aligned with the fresh weight and water content loss, provided electrolyte leakage, oxidative damage, and cell death in the petals [30]. The increasing trend in the activity of nucleases (including DNase and RNase) and proteases during vase life was prevented by 5% HNW [30]. Thus, HNW delayed petal senescence by reducing ROS accumulation and the initial activities of senescence-associated enzymes [30].

In daylily (*Hemerocallis fulva* L.) cultivar ‘Dawuzui’, HRW is used for preharvest treatment not only to increase bud yield, but also to maintain redox homeostasis by suppressing the gathering of O_2_^•−^ and H_2_O_2_ in daylily buds under conditions of cold storage [31]. It prevents daylily bud sepal from browning during cold storage because it enhances membrane function, maintains fatty acid ratio, and reduces lipid peroxidation extension [31]. Moreover, the increasing total phenolics and the decreasing polyphenol oxidase activity also provide for the alleviation of bud browning [31].

In marigolds (*Tagetes erecta* L.), the use of 50% HRW showed physiological changes such as increasing root number and length of its explants [32]. Compared with the control, the use of hydrogen-rich water extended polyphenol oxidase, peroxidases, and indoleacetic acid oxidase activity [32]. Hydrogen gas promotes adventitious floral explant-root development by relatively increasing water content, metabolic constituents, rooting-related enzymes, and simultaneously maintaining cell membrane integrity (Table 1) [32].

Magnesium hydride (MgH_2_), which is a suitable solid-state hydrogen source with high-capacity storage (7.6 wt%), was first applied as a hydrogen generation source with 98% purity and 0.5–25 μm size for floral postharvest preservation in cut carnation flowers [33]. Combining MgH_2_ and citrate buffer solution could greatly increase efficiency compared to that of MgH_2_ solutions in water [33]. The production and hydrogen residence time in solution were increased when compared with HRW [33]. Redox homeostasis was re-established and the progressing transcripts of representative senescence-associated genes, together with *DcbGal* and *DcGST1*, partly disappeared [33]. In contrast, the considered responses were blocked by the inhibition of endogenous H_2_S with hypotaurine and H_2_S collectors [33]. These results confirmed that MgH_2_-supplying H_2_ could extend cut carnation vase life via H_2_S signaling, which could be a possible application of hydrogen-releasing methods in floral postharvest [33].

Endogenous ethylene production and ethylene gene expression in biosynthesis and signaling pathways were studied to determine the link between H_2_ and ethylene during the senescence of cut roses [27]. The addition of exogenous ethylene to ethephon increased the senescence of cut roses, with 100 mg L^−1^ ethephon presenting the most obvious senescent phenotype [27]. The study of HRW (1%) indicated the best vase life quality by reducing ethylene production [27]. It decreased 1-aminocyclopropene-1-carboxylate (ACC) accumulation, as well as ACC synthase (ACS) and ACC oxidase (ACO) activities [27]. It also produced *Rh-ACS3* and *Rh-ACO1* expression in ethylene biosynthesis [27]. HRW increased the transcripts of ethylene receptor genes *Rh-ETR1* from day 4 to day 6 in the blooming period and suppressed *Rh-ETR3* at day 8 after harvest in the senescence phase [27]. The effect of HRW on *Rh-ETR1* and *Rh-ETR3* expression still existed when ethylene production was compromised by adequately adding exogenous ethylene in HRW-treated cut rose petals, and HRW directly repressed the protein level of *Rh-ETR3* in a transient expression assay [27].

## 3. The Potential Observation Using Hydrogen Tools in Floral Preservative Solution

Currently, the COVID-19 pandemic has impacted the global economy, including the flower industry. Thus, the preservative solution not only prolongs flower life but also prevents the substantial drop in prices of exporting flowers. Floral preservative solutions have been widely used by growers, florist sellers, and customers to extend vase life and maintain the quality of cut flowers [9]. Preservative solutions have many advantages, such as reducing bacterial agents in the vase, increasing water uptake, and balancing the carbohydrate requirement for metabolic cycle activities of cut flowers [9,34,35]. Floral preservative solutions can be separated into two types: chemical solutions and eco-friendly solutions [8]. Chemical preservative solutions, such as aluminum sulfate (Al_2_(SO_4_)_3_), aminooxyacetic acid (AOA), benzyladenine (C_12_H_11_N_5_), calcium, calcium nitrate, calcium dichloride (CaCl_2_), chlorine compounds (sodium hypochlorite, sodium dichloroisocyanurate, chlorine dioxide (ClO_2_), cobalt chloride (CoCl_2_)), hydroquinone (HQ), 8-hydroxyquinoline sulfate (8-HQS), silver thiosulfate (STS), silver nitrate (AgNO_3_), isothiazolinone, and quaternary ammonium chloride, can extend vase life, develop flower openings, and recover flower stem and size or petal color by balancing osmotic regulation [8,36]. Eco-friendly solutions can coincide with various factors such as prolonged vase life, controlled water uptake, and prevention of bacterial growth [8]. The H_2_ solution was divided into an eco-friendly preservative. H_2_ is known to affect cellular functions in plant cells [37]. HRW can extend the vase life of cut flowers, including carnations [30,38], roses [25], lisianthus [26], and lilies [28]. A minor drawback of H_2_ in HRW is the residence time, which is commonly shorter than its present half-life in water of approximately 100 min [30]. However, H_2_ application is advantageous in that it promotes the formation of nanobubbles with high internal pressure and negatively charged surfaces, which can increase the residence time and solubility in liquid [39].

HNW may have broad applications, not only in supporting human health care but also in extending the quality of floral life. HNW reduced ROS accumulation induced by senescence, thereby maintaining membrane integrity; HNW induces the initial inhibition of nuclease and protease activity, which may partially alleviate cell death, delay senescence, and prolong the life of flowers. In conclusion, molecular hydrogen can be applied to the floral industry for extending floral vase life, as long as the supplied tools of HNW-mediated H_2_ show increasing availability of H_2_, which has been a powerful tool in horticulture. Furthermore, they reduce ROS accumulation and inhibit the activities of proteases and nucleases.

Hydrogen is most frequently stored in tanks as gas or liquid for small-scale mobile and stationary applications. In general, geological storage is the best choice for large-scale and long-term storage, whereas tanks are more suitable for short-term and small-scale storage. The cost-benefit analysis of H_2_ application in floral preservatives postharvest does not sufficiently compare chemical and eco-friendly solutions. Although renewable H_2_ is expensive, innovative technologies, such as water electrolysis, are estimated to reduce production costs. Thus, the estimated cost of H_2_ application is mainly dependent on labor costs under economic conditions [40]. However, chemical effects in physiological situations have not been established. There are various ways to regulate the senescence of cut flowers, such as NO, calcium ion (Ca^2+^)/calmodulin (CaM) [41], sodium hypochlorite + aminoisobutyric acid + 1-methylcyclopropene (ClAM) [29], and sucrose + ClO_2_ [42]. When Ca^2+^ chelators, Ca^2+^ channel inhibitors, and CaM antagonists are applied, the promoting effects of NO on vase life are blocked [41]. The Ca^2+^ channel inhibitor nifedipine itself negatively impacts fresh-keeping by inhibiting endogenous Ca^2+^ [41]. Hydrogen solution can be preferred over other methods [40,43]. Hydrogen solution is active against a broad range of micro-organisms, including bacteria, yeasts, and fungi, and is eco-friendly [40,43]. We expect that in the period of low-carbon agriculture, H_2_ presents unique renewable and eco-friendly solutions for the environment and people, while also reducing greenhouse gas emissions on ignition.

## 4. Further Prospects for Hydrogen Treatment in the Floral Industry in Korea

In Korea, the Korea Seed & Variety Service noted that, of the 7731 crops filed and registered to date, flowers constituted 4123 representing 53% of the total registered crops [44]. Since the 1980s, floral genetic resources have been focused on culturing experiments with floral varieties such as chrysanthemum, rose, trumpet lily, and carnation [44]. In the following ten years, global agricultural products have enabled the introduction of new flower varieties and seedlings for export [44]. In 1995, Korea joined the International Union of the Production of New Varieties of Plants (UPOV), which included various studies on breeding and high-quality seedlings of chrysanthemums, roses, lilies, carnations, hibiscus, and gerberas [44]. During the 2000s, breeding technology was stabilized leading to many new varieties, increasing the ingression rates of chrysanthemums, orchids, and roses from 1% in 2000 to 5.8% in 2008, and 27.3% in the 2010s [44]. In Korea, there are some representative domestic varieties of breeding samples such as “Baekma” (chrysanthemums), “Deep purple” (rose), “Woori tower” (lily), and “Shiny gold” (freesia) [44].

For the Korean floriculture industry, it could be beneficial to use HRW and HNW, which are cheap, eco-friendly, non-toxic to humans, and provide a long life for cut flowers. H_2_ can be linked to plant stresses, such as temperature, heavy metals, salinity, and light stress, which is promising for the use of H_2_ treatment to delay postharvest senescence (Table 2) [44]. However, the effects of HRW are visible during postharvest if the plants are also treated at preharvest [45]. HRW has a short residence time with a half-life of approximately 100 min in water [30]. HNW diminishes ROS accumulation and is associated with delayed response senescence and extended flower vase life. H_2_ is approved by other industries [46], which are similar in that its creation, storage, and transport costs will become cheaper, combined with an attractive sense in agricultural production [40,44]. H_2_ treatments, which are representative solutions such as HRW or HNW, could be associated with other treatments, including fertilizers, also resulting in lower costs in the floral industry. Even if the current costs are excessive, the application of H_2_-based treatments is likely to be efficient in the future, and these may be extremely promising for a range of postharvest uses [40,44]. Although few studies exist on using hydrogen treatment in the floral industry, H_2_ can be used in solution or donor molecule forms and can improve the quality of floral postharvest. In postharvest solutions, especially in the floral industry, the use of many H_2_-based treatments is expected to investigate the optimization of H_2_ delivery methods and provide solutions that are suitable to the crop being used. This is a safe, eco-friendly, and easy way of using H_2_ and its form for application in the floral postharvest and horticultural industries in Korea and internationally. Further investigation of H_2_-based treatments in Korea could expand, as could the development of innovative tools, which would be re-affirmed by cost-benefits analysis.

## 5. Conclusions

**Figure 2:**
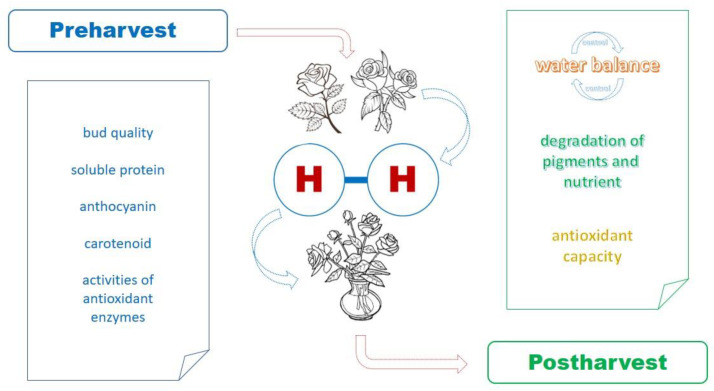
The physiological diagram for the study of floral senescence causes and hydrogen solution.

This paper considered eco-friendly tools to improve cut flower vase life and is intended not only to help scientists, especially florists, to understand hydrogen technologies but also to provide an overview of steps for keeping cut flowers with a long vase life. The use of hydrogen solutions for cut flowers must be investigated and developed (Figure 2), and innovative tools should be provided based on their suitability for the environment and human health. These hydrogen-based treatments should be considered and investigated for their benefits related to Korean floral postharvest.

## Figures and Tables

**Table 1:** An overview on the hydrogen forms used for floral treatments.

Hydrogen Forms	Floral Treatments	Utilization Treatment Parameters	Results	References
**Hydrogen rich water** **(HRW)**	Cut rose (*Rosa hybrida* ‘Movie star’) Daylily (*Hemerocallis fulva* L.) cultivar ‘Dawuzui’ Marigold (*Tagetes erecta* L.) explants	1% HRW Preharvest: 0.8 μmol L^−1^ H_2_ 50% HRW	Development of beneficial bacteria abundances on the stem-end cut surface. Improvement of yield and quality. Induced root development	[20] [31] [32]
**Hydrogen nanobubble water** **(HNW)**	Carnation (*Dianthus caryophyllus* L.) cultivar ‘Pink Diamond’	5% HNW	Development of the effective concentration and residence time of H_2_ in water for extending vase life.	[30]
**Magnesium hydride** **(** **MgH_2_** **)**	Carnation (*Dianthus caryophyllus* L.) cultivar ‘Pink Diamond’ Cut rose (*Rosa hybrida* ‘Carola’)	MgH_2_ (0.1 g L^–1^) with citrate MgH_2_ (0.001 g L^–1^) with H_2_-releasing donor	MgH_2_-prolonged vase life of cut carnation flowers via increasing GST expression. Re-establishing redox homeostasis to extend vase life	[33] [21]

**Table 2:** Collection of H_2_ treatments (Hydrogen-rich water—HRW, Hydrogen nanobubble water—HNW) for cut flowers in postharvest. H_2_ concentrations are converted from the information given in the reference papers [43].

Flower Investigation	Treatment	Result	Reference
Rose ‘Movie star’	1% HRW (best in 0.00235 mM H_2_)	Less flower senescence. Investigated by ethylene metabolism.	[27]
Lily (*Lilium* spp.) and rose (*Rosa hybrid* L.)	Lily: 0.5% HRW (2.25 µM H_2_) and 1% (4.5 µM H_2_); Rose: 50% HRW (0.225 mM H_2_)	Extended vase life. Greater flower diameter. Reduced oxidative stress.	[25]
Lily (*Lilium* ‘Manissa’)	1% HRW (0.0022 mM H_2_) and 150 μM sodium nitroprusside (SNP)	Improved flower freshness. ATP synthase CF1 alpha subunit (AtpA) up-regulated.	[28]
Lisianthus (*Eustoma grandiflorum*)	HRW (0.078 mM H_2_)	Vase life prolonged. Redox maintained as reducing oxidative stress.	[26]
Carnation (*Dianthus* *caryophyllus* L.).	Hydrogen nanobubble water (5% HNW): best in 0.025 mM H_2_	Less senescence leading to prolonged vase life. Minimized oxidative stress.	[30]

## Data Availability

Not applicable.
